# Effects of Microbial Fermented Feed on Serum Biochemical Profile, Carcass Traits, Meat Amino Acid and Fatty Acid Profile, and Gut Microbiome Composition of Finishing Pigs

**DOI:** 10.3389/fvets.2021.744630

**Published:** 2021-11-04

**Authors:** Xiaopeng Tang, Xuguang Liu, Kai Zhang

**Affiliations:** ^1^State Engineering Technology Institute for Karst Desertfication Control, School of Karst Science, Guizhou Normal University, Guiyang, China; ^2^College of Animal Science, Shanxi Agricultural University, Jinzhong, China

**Keywords:** fermented complete feed, finishing pigs, carcass traits, meat quality, gut microbiome, amino acid, fatty acid

## Abstract

Microbial fermented feed is an important part of feed industry, while little research has focused on the solid-state fermentation of complete feed. Herein, the purpose of the present study was to investigate the effects of fermented complete feed (FCF) on the growth performance, biochemical profile, carcass traits, meat proximate composition, meat amino acid and fatty acid profile, and gut microbiome composition of finishing pigs, thereby providing references for the application of FCF in animal production. Twenty Duroc × Landrace × Yorkshire pigs with an average body weight (BW) of 48.74 ± 1.49 kg were divided randomly into control group (pigs received a basal diet, CN, *n* = 10) and FCF group (pigs fed with FCF, *n* = 10). The experiment lasted for 60 days. FCF improved the growth performance, which was indicated by a significantly increased final BW, average daily gain and average daily feed intake, and a significantly decreased feed-to-gain ratio. FCF improved biochemical profile, which was indicated by a higher alkaline phosphatase, glucose, immunoglobulin G, immunoglobulin M, superoxide dismutase, and total antioxidant capacity content. Pigs that received FCF had better carcass traits and meat quality than did pigs that received basal diet, which was indicated by a higher carcass length, crude protein content, lysine content, Glu content, C18:ln9c, C18:2n6c, C20:4n6, and unsaturated fatty acid content and a lower average back-fat thickness, C18:0, and saturated fatty acids. FCF significantly reduced the relative abundances of presumably pathogenic bacteria of phylum *Proteobacteria* and genus *Escherichia–Shigella* and enhanced the relative abundances of likely beneficial bacteria of phylum *Firmicutes* and genus *Clostridium*. In summary, FCF had a certain effect on the improvement of growth performance, serum biochemical profile, carcass traits, meat proximate composition, amino acid and fatty acid profile, and gut microbiome composition of finishing pigs.

## Introduction

Nowadays, the researches of antibiotic substitutes, such as probiotics (1), antimicrobial peptides (2), Chinese herbal medicine additives (3), plant-derived phytochemicals (4), functional amino acids (AAs) (5), organic acids (6), and other functional additives (7, 8) without potential threats have become a hot issue in the field of animal nutrition. Microbial fermented feed (MFF), including liquid fermentation feed and solid-state fermentation feed, is an important part of feed industry, which has become an effective strategy to reduce or no use of antibiotics in animal feed (9). Previous studies have demonstrated that MFF not only can improve growth performance, but also can improve animal health (9–11). For example, Chen et al. (10) reported that Landes geese fed with fermented liquid feed had a positive effect on growth performance, relative organ weight, intestinal microflora, and organ antioxidant status. However, previous studies were mainly focused on the liquid fermentation feed (10, 12) and solid-state fermentation of single feed ingredients, such as cottonseed meal (13), soybean meal (14), and cassava residue (15); little research has focused on the solid-state fermentation of complete feed, especially the effect of fermented complete feed (FCF) on growth performance, and intestinal microbiome composition of pigs has not been investigated in detail.

At present, because of the excessive attention to the breeding of growth rate, back-fat thickness, and lean meat percentage in the long-term breeding process, the quality of pork has been declining. Thus, improving meat quality by means of nutrition is a current hot topic in animal research. Previous studies have found that many strategies can improve meat quality (16–18). Among them, nutritional strategies are an effective way to improve meat quality. For example, Ornaghi et al. (19) reported that beef cattle fed with a mixture of natural additives significantly influenced the pH, shear force, tenderness, and lipid oxidation of meat. Cheng et al. (20) reported that intrauterine growth–retarded pigs fed with resveratrol could improve meat quality, muscular antioxidant capacity, lipid metabolism, and fiber-type composition of meat. MFF as an important part of feed industry can also improve meat quality. Zhu et al. (21) showed that pigs fed with a mixture diet containing 80% basal diet and 20% fermented feed can increase the meat color and tenderness of longissimus dorsi and improve the meat quality of finishing pigs. However, the effect of FCF on meat quality of pigs has not been investigated in detail. Therefore, the present study aimed to study the effects of FCF on growth performance, biochemical profile, carcass traits, meat proximate composition, AA and fatty acid profile, and gut microbiome composition of finishing pigs, thereby providing references for the application of FCF in animal production.

## Materials and Methods

### The Preparation of FCF

The basal diet was prepared according to the feeding standard of swine (NY/T 65-2004) to meet the nutrient requirement for finishing pigs (Table 1). The solid-state fermentation process was as follows: *Lactobacillus plantarum, Candida utilis, Bacillus subtilis*, and *Aspergillus niger* were selected as the starting strains, and the regeneration and propagation were carried out under the corresponding medium and culture conditions. When *L. plantarum* and *B. subtilis* content reached 1 × 10^8^ colony-forming units (CFU)/g (1–2 days), the *C. utilis* content reached 1 × 10^8^ CFU/g (3–5 days), and *A. niger* content reached 1 × 10^7^ CFU/g (3–5 days), the fermentation microbial liquid was mixed with a volume ratio of *L. plantarum*: *C. utilis*: *B. subtilis*: *A. niger* = 1:3:3:2. The basal diet with a moisture content of 30% was inoculated with 0.5% of fermentation microbial liquid and anaerobic fermented at 24°C to 34°C for 72 h. After fermentation, crude protein (CP, 16.92%), crude fiber (CF, 3.27%), *L. plantarum* (1.5 × 10^8^ CFU/g), *C. utilis* (2.1 × 10^6^ CFU/g), and *B. subtilis* (3.0 × 10^8^ CFU/g) contents were measured.

**Table 1:** Composition and nutrient level of basal diet.

**Ingredient**	**Content (%)**	**Nutrient levels^b^**	**Content (%)**
Corn	76.3	Digestible energy, MJ/kg	14.26
Soybean meal	16.5	Crude protein	15.89
Soybean oil	0.56	Crude fiber	3.45
Corn starch	2.44	Crude fat	5.94
CaHPO_4_	0.65	Calcium	0.62
Calcium carbonate	1.08	Total phosphorus	0.42
Sodium chloride	0.43	Available phosphorus	0.20
Lysine	0.16		
Premix^a^	1.75		
Total	100		

a *Premix for 1 kg of complete diet contained Cu as copper sulfate, 10 mg; Fe as iron sulfate, 100 mg; Se as sodium selenite, 0.30 mg; Zn as zinc oxide, 100 mg; Mn as manganese oxide, 10 mg; vitamin D 3, 386 IU; vitamin A as retinyl acetate, 3,086 IU; vitamin E as d-tocopherol, 15.4 IU; vitamin K as menadione sodium bisulfate, 2.3 mg; vitamin B_2_, 3.9 mg; calcium pantothenate, 15.4 mg; niacin, 23 mg; vitamin B_12_, 15.4 mg*.

b *DE was a calculated value. The other nutrient levels were measured values*.

### Animals and Experimental Design

Twenty Duroc× Landrace× Yorkshire pigs with an average body weight (BW) of 48.74 ± 1.49 kg were purchased from Shanxi ShouKang Farming Cooperatives (Taiyuan, China) and were divided randomly into control group (pigs received a basal diet, CN) and FCF group (pigs received FCF). Each treatment contains 10 replicates. For simplicity of operation, the same basic diet was used throughout the trial period. Of course, in the actual production, we would not use only one type of feed, which cannot cover adequately the nutritional requirements of each growth phase. All pigs were individually housed in stainless steel cages equipped with a feeder and a nipple drinker, and all pigs were fed three times per day and had *ad libitum* access to water (22). The room lighting was natural, and the room temperature was maintained at an ambient temperature range of 25–28°C. The experiment lasted for 60 days.

Pigs were weighed at the first day and the last day of the experiment, and the initial BW and final BW of pigs were recorded, to calculate the average daily gain (ADG) of pigs. The feed consumption was recorded weekly to calculate the average daily feed intake (ADFI) of pigs. Feed-to-gain (F/G) ratio was calculated by ADG and ADFI, where F/G ratio = ADFI/ADG.

### Sample Collection

At the end of the feeding trial, the diet was removed 12 h before slaughter. All pigs were anesthetized with an injection of sodium pentobarbital (50 mg kg^−1^ BW), and then 10 mL of blood was collected aseptically in tubes from the jugular vein. After blood collection, all pigs were killed by exsanguinations. Serum samples were separated from the blood after centrifugation at 3,000 g for 10 min at 4°C and stored at −20°C for serum biochemical indices and antioxidant indices measurement (*n* = 10). Jejunum and cecum digesta samples were collected from the middle jejunum and cecum and then immediately frozen in liquid nitrogen and stored at −80°C for microbiota analysis (*n* = 3). After the removal of the head, feet, tail, and internal organs, the carcass was weighed to determine carcass yield (dividing the carcass weight by live BW) and then bisected laterally into two parts. The carcass weight, dressing percentage, and back-fat thickness were evaluated following the Chinese Agriculture Industry Standard (NY-T825-2004). The left side of the carcass was cut between the 4th and 5th ribs, the 11th and 12th ribs, and the last rib and first lumbar vertebrae to measure the back-fat thickness (*n* = 10), and the longissimus dorsi muscle (LDM) area was measured between the 12th and 13th ribs (*n* = 10). LDM tissues with subcutaneous fat removed were collected from the right side of each carcass and stored at −20°C for the analysis of CP, crude fat, AA, and fatty acid composition (*n* = 10).

### Serum Biochemical Profile Detection

Serum biochemical indices, including alanine aminotransferase (ALT), aspartate aminotransferase (AST), alkaline phosphatase (ALP), total protein (TP), albumin (ALB), globulin (GLO), urea, glucose (GLU), total cholesterol (TC), triglyceride (TG), immunoglobulin A (IgA), immunoglobulin G (IgG), and immunoglobulin M (IgM) were determined with automatic blood analyzer (Mindray BS-420) in accordance with the manufacturer's instructions (Shenzhen Mindray Bio-Medical Electronics Co., Ltd., Shenzhen, China).

### Serum Antioxidant Property

Serum antioxidant indices, including superoxide dismutase (SOD), total antioxidant capacity (T-AOC), malonaldehyde (MDA), were measured used SOD assay kit (A001-3), T-AOC assay kit (A015-1), and MDA assay kit (A003-2), respectively (Nanjing Jiancheng Bioengineering Institute, Nanjing, China) according to the instructions of the manufacturer.

### Assessment of Meat Quality

Meat quality was assessed by determining pH and drip loss of the LDM. A piece of LDM was used to determine drip loss as previously described (23). The remaining section of LDM was used to determine pH at 45 min (pH_45_ min) and 24 h (pH_24_ h) by inserting the electrode into the core of the muscle parallel to the muscle fibers using a handheld pH meter (Russell CD700, Russell pH Ltd., Germany).

### Proximate Composition of Skeletal Muscle

Muscle samples were cut into slices, dried in a vacuum-freeze dryer, and then ground into powder. The crude protein, crude fat, and AA and fatty acid content were determined according to AOAC (24). The AA profiles were analyzed using an automatic AA analyzer (L-8800; Hitachi, Tokyo, Japan) according to the manufacturer's instructions, and the contents of AA were expressed as mg/g wet tissue. The fatty acid profiles were analyzed using an automatic fatty acid analyzer (Shimadzu GC-2010 plus, Japan) according to the manufacturer's instructions.

### DNA Extraction, 16s Ribosomal RNA Amplicon, and Sequencing

Total genome DNA from jejunum digesta and cecum digesta was extracted using QIAamp DNA Stool Mini Kit (Qiagen GmbH, Hilden, Germany) following the manufacturer's instructions. The extracted genomic DNA concentration and purity were determined using a NanoDrop 2000 UV-vis spectrophotometer (Thermo Fisher Scientific, Waltham, MA, USA), and the DNA integrity was checked using 1% agarose gel electrophoresis. Illumina MiSeq sequencing and bioinformatics analyses were performed by a commercial company (Biomarker, Beijing, China). The V3–V4 region of the bacterial 16S rRNA gene was amplified to define the bacterial composition and abundance by PCR using bacterial universal primers. The PCR amplicon products were separated on 2% agarose gels, purified, pooled at equimolar concentrations, and paired-end sequenced on an Illumina MiSeq platform according to the standard methods.

### Microbiome Analysis

High-quality sequences were uploaded to QIIME (version 1.7.0) for further study. FLASH (v1.2.7) was used to splice reads of each sample through overlap to obtain splicing sequence (Raw Tags), Trimmomatic (v0.33) was used in the Raw Tags to get “clean tags,” and UCHIME software (v4.2) was used to identify and remove chimera sequences to obtain the final valid data (effective tags). Then, effective tags were clustered into operational taxonomic units (OTUs) using UCLUST in QIIME (version 1.7.0) at 97% sequence identity. The OTUs were annotated based on Silva (bacteria) and UNITE (fungi) taxonomy databases. Then, the microbiome composition of each sample was calculated at each level (phylum, class, order, family, genus, and species), and QIIME software was used to generate species abundance tables at different classification levels. Alpha diversity (ACE index, Chao1 index, Shannon index, and Simpson index) was calculated by Mothur (v1.30) software. The linear discriminant analysis effect size (LEfSe) analysis was conducted by LEfSe software.

### Statistical Analysis

Serum biochemical indices, carcass traits, meat quality, meat AA and fatty acid composition were subjected to the unpaired *t*-test using SPSS 21.0 software (SPSS, Inc., Chicago, IL, USA). The data are presented as the means ± SE (standard error). The alpha diversity indices, relative species abundances, and overall composition (at phyla and genera levels) of gut microbial communities were analyzed using the Kruskal–Wallis test. LEfSe was used to identify different taxa microbes among lines using default parameters. *p* < 0.05 was taken to indicate statistical significance.

## Results

### Growth Performance

The effects of FCF on the growth performance of pigs are presented in Table 2. It showed that pigs fed with FCF had a significant effect on growth performance, which was indicated by significantly (*p* < 0.05) increased final BW, ADG, and ADFI and significantly (*p* < 0.05) decreased of F/G ratio in FCF group.

**Table 2:** Effects of fermented complete feed on growth performance of pigs.

**Item**	**CN**	**FCF**	***P*** **value**
Initial body weight (kg)	48.52, 1.53	48.97, 1.34	0.327
Final body weight (kg)	100.34, 4.48	104.56, 7.76	0.031
ADG (kg/d)	0.86, 0.05	0.93, 0.01	0.045
ADFI (kg/d)	2.72, 0.11	2.85, 0.21	0.028
F/G ratio	3.16, 0.15	3.06, 0.19	0.037

*Values are expressed as means ± SE, n = 10; CN, control group (pigs received a basal diet); FCF, fermented complete feed group (pigs received fermented complete feed)*.

### Serum Biochemical Profile

The effects of pigs fed with FCF on the serum biochemical indices are presented in Table 3. Compared with the CN group (pigs fed with basal diet), the FCF group (pigs fed with FCF) had a higher (*p* < 0.05) serum ALP, GLU, IgG, and IgM levels.

**Table 3:** Effects of fermented complete feed on serum biochemical indices of pigs.

**Item**	**CN**	**FCF**	***P*** **-value**
AST (U/L)	21.18, 2.28	22.78, 3.25	0.394
ALT (U/L)	43.18, 2.02	45.92, 4.95	0.387
ALP (U/L)	98.99, 3.93	118.27, 16.27	0.033
TP (g/L)	57.79, 5.73	63.62, 8.43	0.237
Albumin (g/L)	24.26, 2.86	26.24, 3.42	0.350
Globulin (g/L)	33.53, 6.47	37.37, 7.42	0.536
Urea (mmol/L)	6.25, 0.45	5.57, 0.42	0.370
Glucose (mmol/L)	4.72, 0.37	5.37, 0.46	0.041
Cholesterol (mmol/L)	2.28, 0.13	2.06, 0.20	0.072
Triglyceride (mmol/L)	0.62, 0.07	0.70, 0.10	0.217
IgA (g/L)	1.33, 0.03	1.36, 0.03	0.174
IgG (g/L)	19.57, 0.42	20.34, 0.44	0.021
IgM (g/L)	2.36, 0.09	2.49, 0.06	0.018

*Values are expressed as means ± SE, n = 10. CN, control group (pigs received a basal diet); FCF, fermented complete feed group (pigs received fermented complete feed); AST, aspartate aminotransferase; ALT, alanine aminotransferase; ALP, alkaline phosphatase; TP, total protein; IgA, immunoglobulin A; IgG, immunoglobulin G; IgM, immunoglobulin M*.

### Serum Antioxidant Property

The effects of FCF on serum antioxidant property are presented in Table 4. Compared with the CN group, the pigs fed with FCF had a higher (*p* < 0.05) serum SOD and T-AOC levels. But there was no difference about MDA between the CN and FCF groups.

**Table 4:** Effects of fermented complete feed on serum antioxidant property of pigs.

**Item**	**CN**	**FCF**	***P-*** **value**
SOD (U/mL)	42.40, 2.69	46.39, 1.67	0.023
T-AOC (U/mL)	11.64, 0.64	13.85, 1.65	0.024
MDA (nmol/mL)	5.45, 0.44	4.96, 0.62	0.180

*Values are expressed as means ± SE, n = 10. CN, control group (pigs received a basal diet); FCF, fermented complete feed group (pigs received fermented complete feed); SOD, superoxide dismutase; T-AOC, total antioxidant capacity; MDA, malonaldehyde*.

### Carcass Traits

The effects of FCF on carcass traits of pigs are shown in Table 5. Compared with the CN group, pigs that received FCF had significantly (*p* < 0.05) increased carcass length (129.83 vs. 129.33 cm) and significantly (*p* < 0.05) decreased average back-fat thickness (3.62 vs. 3.68 cm). Although no statistically significant differences (*P* > 0.05) were observed in carcass weight, slaughter rate, and loin eye area between the CN and FCF groups.

**Table 5:** Effects of fermented complete feed on carcass traits of finishing pigs.

**Items**	**CN**	**FCF**	***P-*** **value**
Carcass weight (kg)	94.80, 0.64	95.10, 1.134	0.341
Slaughter rate (%)	72.59, 1.31	73.10, 1.87	0.265
Carcass length (cm)	128.33, 0.33	129.83, 1.74	0.025
Average back-fat thickness (cm)	3.68, 0.06	3.62, 0.07	0.036
Loin eye area (cm^2^)	43.2, 2.14	44.6, 0.89	0.487

*Values are expressed as means ± SE, n = 10. CN, control group (pigs received a basal diet); FCF, fermented complete feed group (pigs received fermented complete feed)*.

### Meat Quality

The effects of the FCF on meat quality of pigs are presented in Table 6. It showed that pigs fed with FCF had a higher (*p* < 0.05) crude protein content (22.37 vs. 21.67%) compared with pigs fed with basal diet. However, there was no significant difference in drip loss, pH_45_ min, pH_24_ h, and fat in LDM between CN and FCF groups.

**Table 6:** Effects of fermented complete feed on meat quality of finishing pigs.

**Items**	**CN**	**FCF**	***P*** **value**
Drip loss (%)	2.20, 0.05	2.21, 0.06	0.524
pH_45min_	6.67, 0.03	6.73, 0.01	0.451
pH_24h_	6.12, 0.14	6.24, 0.21	0.214
Crude protein (%)	21.67, 0.39	22.37, 0.59	0.024
Fat (%)	4.94, 1.12	5.06, 0.20	0.651

*Values are expressed as means ± SE, n = 10. CN, control group (pigs received a basal diet); FCF, fermented complete feed group (pigs received fermented complete feed)*.

### Meat Amino Acid Composition

The effects of the FCF on AA contents of LDM are presented in Table 7. Pigs that received FCF had a higher (*p* < 0.05) Lys content (1.86 vs. 1.74%) and Glu content (3.94 vs. 3.53%) compared with pigs fed with basal diet, whereas there was no difference of other AAs between the two groups.

**Table 7:** Effects of fermented complete feed on meat amino acid content in longissimus dorsi muscle of finishing pigs (mg/g).

**Amino acids**	**CN**	**FCF**	***P*** **-value**
Asp	2.04, 0.07	2.14, 0.14	0.851
Glu	3.53, 0.11	3.94, 0.32	0.035
Cys	0.45, 0.02	0.47, 0.01	0.115
Ser	0.81, 0.02	0.89, 0.05	0.403
Gly	0.89, 0.04	0.90, 0.02	0.816
His	1.32, 0.04	1.39, 0.06	0.410
Arg	1.50, 0.03	1.60, 0.09	0.254
Thr	0.94, 0.02	1.02, 0.06	0.265
Ala	1.13, 0.03	1.21, 0.06	0.484
Pro	0.82, 0.03	0.86, 0.03	0.726
Tyr	0.84, 0.01	0.94, 0.05	0.541
Val	1.12, 0.03	1.18, 0.06	0.321
Met	0.94, 0.03	1.02, 0.06	0.587
Ile	0.87, 0.04	0.92, 0.09	0.306
Leu	1.58, 0.05	1.69, 0.14	0.124
Phe	0.68, 0.03	0.71, 0.08	0.166
Lys	1.74, 0.06	1.86, 0.17	0.024
EAA	7.93, 0.26	8.19, 0.66	0.656
FAA	7.59, 0.19	8.16, 0.53	0.381
TAA	21.26, 0.53	22.50, 1.47	0.135

*Values are expressed as means ± SE, n = 10. CN, control group (pigs received a basal diet); FCF, fermented complete feed group (pigs received fermented complete feed); EAA, essential amino acid; DAA, delicious amino acid; TAA, total amino acid*.

### Meat Fatty Acid Composition

The effects of the FCF on fatty acid composition of LDM are presented in Table 8. It showed that pigs that received FCF had a higher (*p* < 0.05) oleic acid (C18:1n9c, 18.721 vs. 17.791 g/kg), linoleic acid (C18:2n6c, 10.163 vs. 9.664 g/kg), arachidonic acid (C20:4n6, 0.342 vs. 0.306 g/kg), and total unsaturated fatty acids (UFAs, 32.004 g/kg vs. 30.361 g/kg) content, as well as had a lower (*p* < 0.05) stearic acid (C18:0, 5.720 vs. 6.177 g/kg) and total saturated fatty acids (SFAs) (15.048 vs. 16.245 g/kg) content compared with pigs fed with basal diet.

**Table 8:** Effects of fermented complete feed on meat fatty acid content in longissimus dorsi muscle of finishing pigs (g/kg).

**Fatty-acids**	**CN**	**FCF**	***P*** **-value**
C10:0	0.020 ± 0.001	0.019 ± 0.001	0.587
C11:0	0.001 ± 0.000	0.001 ± 0.000	0.894
C12:0	0.018 ± 0.002	0.017 ± 0.002	0.482
C14:0	0.293 ± 0.021	0.264 ± 0.028	0.368
C15:0	0.015 ± 0.001	0.013 ± 0.001	0.209
C16:0	9.520 ± 0.369	8.832 ± 0.507	0.158
C16:1	0.879 ± 0.082	0.939 ± 0.090	0.215
C17:0	0.111 ± 0.014	0.106 ± 0.011	0.551
C17:1	0.111 ± 0.014	0.106 ± 0.011	0.365
C18:0	6.177 ± 0.764	5.720 ± 0.137	0.047
C18:1n9c	17.791 ± 0.195	18.721 ± 0.367	0.038
C18:1n9t	1.258 ± 0.022	1.320 ± 0.033	0.354
C18:2n6c	9.664 ± 0.325	10.163 ± 0.520	0.024
C20:0	0.054 ± 0.008	0.046 ± 0.003	0.469
C20:1	0.139 ± 0.019	0.171 ± 0.026	0.356
C20:2	0.158 ± 0.024	0.158 ± 0.019	0.547
C20:3n3	0.072 ± 0.007	0.079 ± 0.007	0.632
C20:3n6	0.043 ± 0.006	0.053 ± 0.002	0.501
C20:4n6	0.306 ± 0.024	0.342 ± 0.026	0.015
C22:0	0.035 ± 0.004	0.030 ± 0.005	0.250
Saturated fatty acids	16.245 ± 0.403	15.048 ± 0.514	0.025
Monounsaturated fatty acids	20.117 ± 1.598	21.208 ± 1.568	0.179
Polyunsaturated fatty acids	10.243 ± 0.975	10.796 ± 0.886	0.463
Unsaturated fatty acids	30.361 ± 1.643	32.004 ± 1.840	0.036

*Values are expressed as means ± SE, n = 10. CN, control group (pigs received a basal diet); FCF, fermented complete feed group (pigs received fermented complete feed)*.

### Gut Microbiota Composition

**Figure 1:**
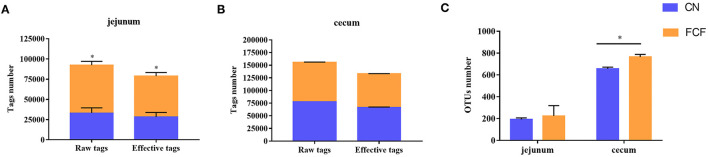
Tag numbers and OTU numbers in each sample (*n* = 3). **(A)** Tag number of jejunum content, **(B)** tag number of cecum content, **(C)** OTU number of jejunum content and cecum content. CN, control group (pigs received a basal diet); FCF, fermented complete feed group (pigs received fermented complete feed). *p < 0.05, compared with the control group.

**Figure 2:**
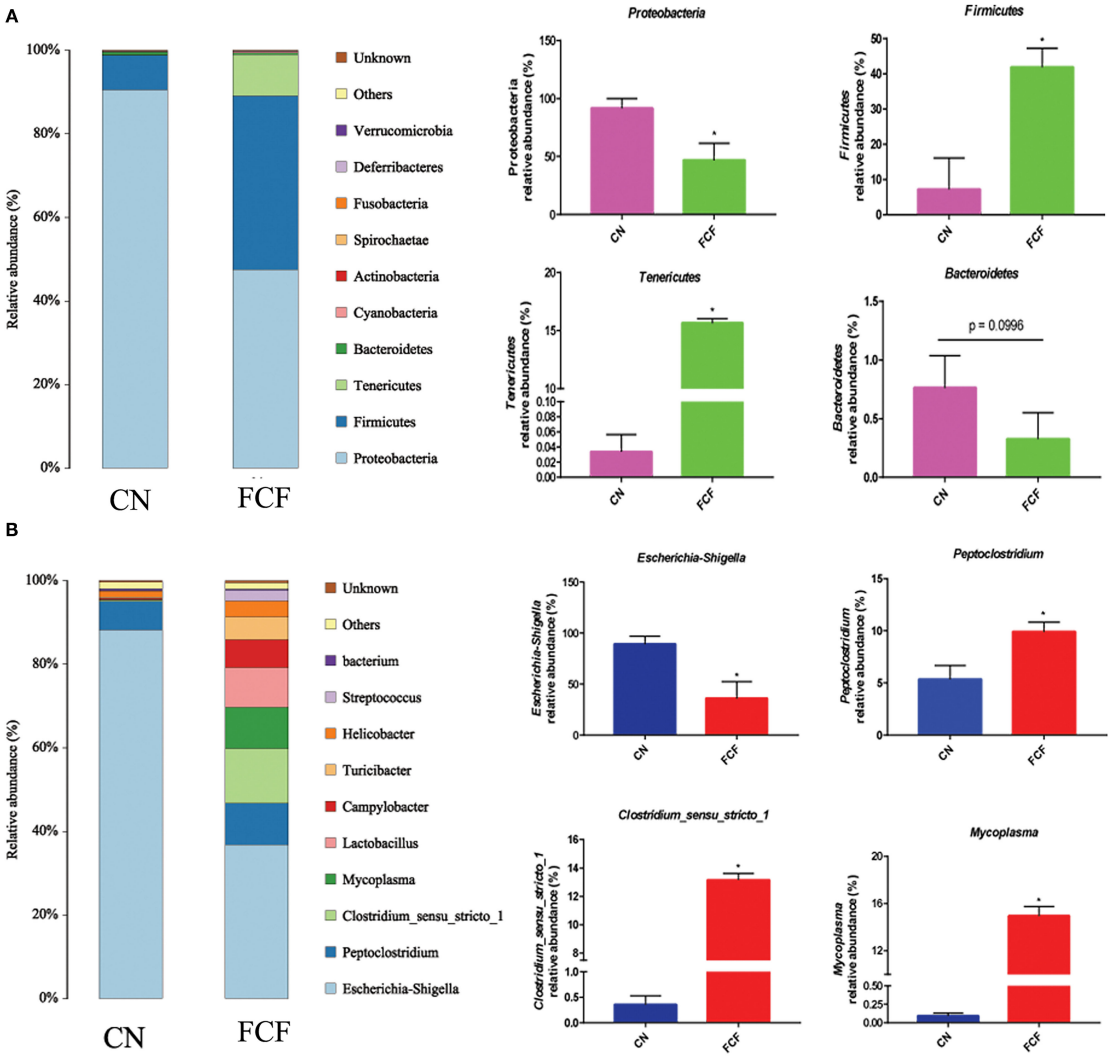
Composition of bacterial communities in jejunum after pigs received fermented complete feed (*n* = 3). **(A)** Relative abundance of bacterial communities at phylum level. **(B)** Relative abundance of bacterial communities at genus level. CN, control group (pigs received a basal diet); FCF, fermented complete feed group (pigs received fermented complete feed). *p < 0.05, compared with the control group.

**Figure 3:**
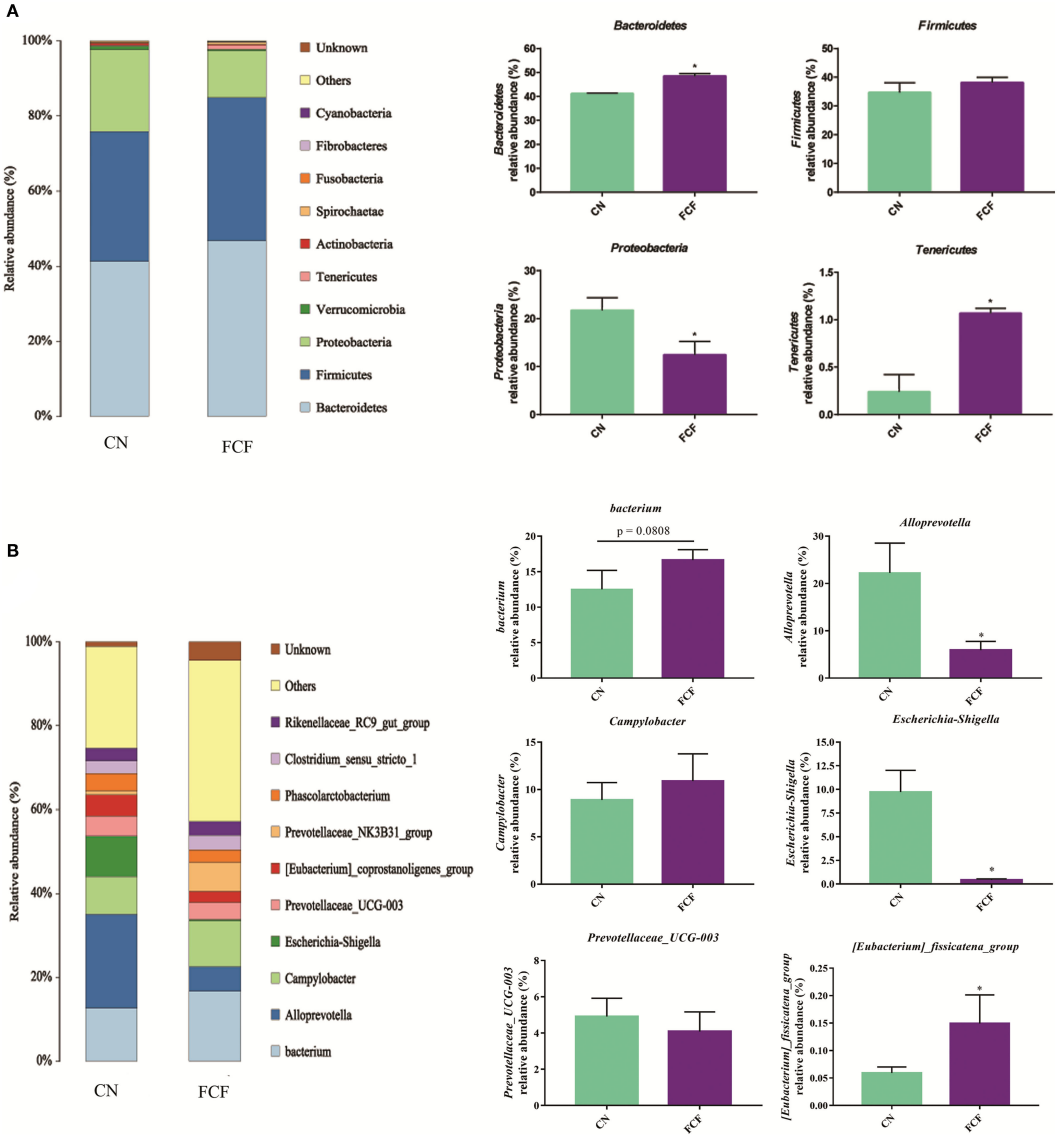
Composition of bacterial communities in cecum after pigs received fermented complete feed (*n* = 3). **(A)** Relative abundance of bacterial communities at phylum level. **(B)** Relative abundance of bacterial communities at genus level. CN, control group (pigs received a basal diet); FCF, fermented complete feed group (pigs received fermented complete feed). *p < 0.05, compared with the control group.

The effects of the FCF on gut microbiota composition are presented in Figures 1–3. A total of 277,077 raw tags and 236,443 effective tags were obtained from jejunum contents, in which FCF group had a higher (*p* < 0.05) raw tags and effective tags than that of the CN group (Figure 1A). The cecum contents had 389,458 raw tags and 399,324 effective tags; however, there was no difference between the CN group and the FCF group (Figure 1B). A total of 1,252 OTUs were found in the jejunum contents, but there was no difference between the CN group and the FCF group; 4,264 OTUs were obtained from cecum contents, and FCF had a higher number of OTUs than the CN group in cecum contents (Figure 1C). At the phylum level, top 10 phyla were identified in the samples from the jejunum contents, of which *Proteobacteria, Firmicutes, Tenericutes*, and *Bacteroidetes* comprised the dominant community members. It showed that FCF significantly (*p* < 0.05) increased *Firmicutes* and *Tenericutes* abundance, while it significantly (*p* < 0.05) decreased *Proteobacteria* abundance in the jejunum (Figure 2A). *Bacteroidetes, Firmicutes, Proteobacteria*, and *Tenericutes* comprised the dominant community members in the samples from the cecum contents. FCF significantly (*p* < 0.05) increased *Bacteroidetes* and *Tenericutes* abundance, while it significantly (*p* < 0.05) decreased *Proteobacteria* abundance in cecum (Figure 3A). At the genus level, *Escherichia–Shigella* abundance in jejunum content was significantly (*p* < 0.05) decreased, and *Peptoclostridium, Clostridium_sensu_stricto_1*, and *Mycoplasma* abundance was significantly increased (*p* < 0.05) after the pigs were fed with FCF (Figure 2B). In cecum content, abundance of *Alloprevotella* and *Escherichia–Shigella* in FCF group was significantly (*p* < 0.05) decreased compared with the CN group, and *[Eubacterium]_fissicatena_group* was significantly (*p* < 0.05) increased; there was an increased tendency of *bacterium* abundance in the FCF group compared with the CN group (Figure 3B).

### Microbiota Diversity

**Figure 4:**
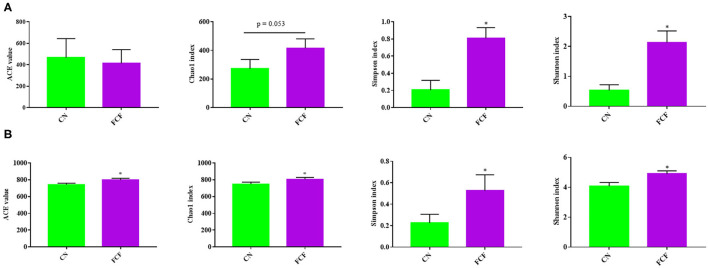
Effect of fermented complete feed on alpha diversity of microbial community in jejunum content **(A)** and cecum content **(B)** of pigs (*n* = 3). CN, control group (pigs received a basal diet); FCF, fermented complete feed group (pigs received fermented complete feed). *p < 0.05, compared with the control group.

Alpha diversity (ACE, Chao1, Shannon, and Simpson index) was calculated to assess the diversity, richness, and phylogenetic diversity of the bacterial community. The results showed that the Shannon index and Simpson index exhibited significant differences (*p* < 0.05) between the CN and FCF groups; the Chao1 index showed a trend for difference (*P* = 0.053) in the jejunum content (Figure 4A), whereas in the cecum content, significant differences (*p* < 0.05) were observed for all alpha diversity values (including ACE, Chao1, Shannon, and Simpson index) between the CN and FCF groups (Figure 4B).

**Figure 5:**
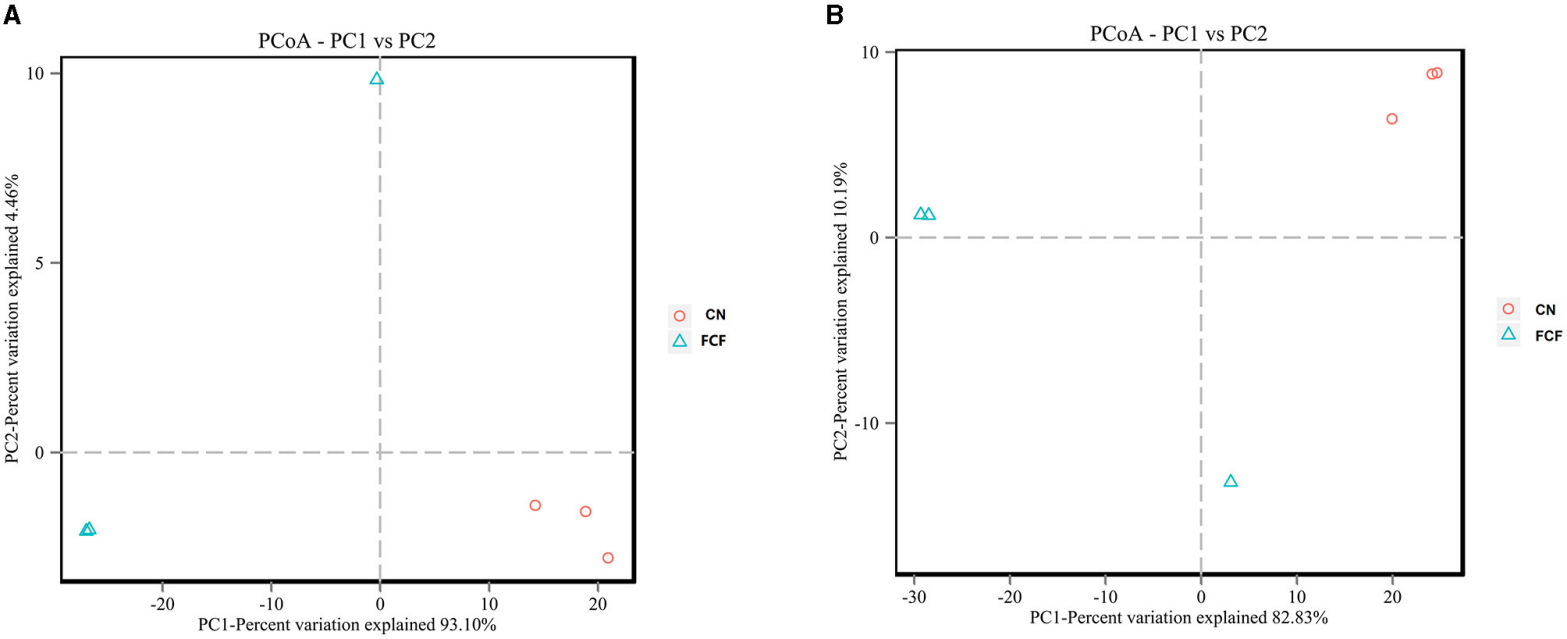
PCoA of the weighted UniFrac distances for CN and FCF groups (*n* = 3). **(A)** Jejunum content and **(B)** cecum content. CN, control group (pigs received a basal diet); FCF, fermented complete feed group (pigs received fermented complete feed).

Weighted UniFrac distances were used to estimate overall differences in beta diversity. The principal coordinates analysis (PCoA) plot of the weighted UniFrac distances showed that CN presented a distinct clustering of microbial community structure, whereas the FCF group showed a scattered distribution structure in both jejunum content (Figure 5A) and cecum content (Figure 5B).

**Figure 6:**
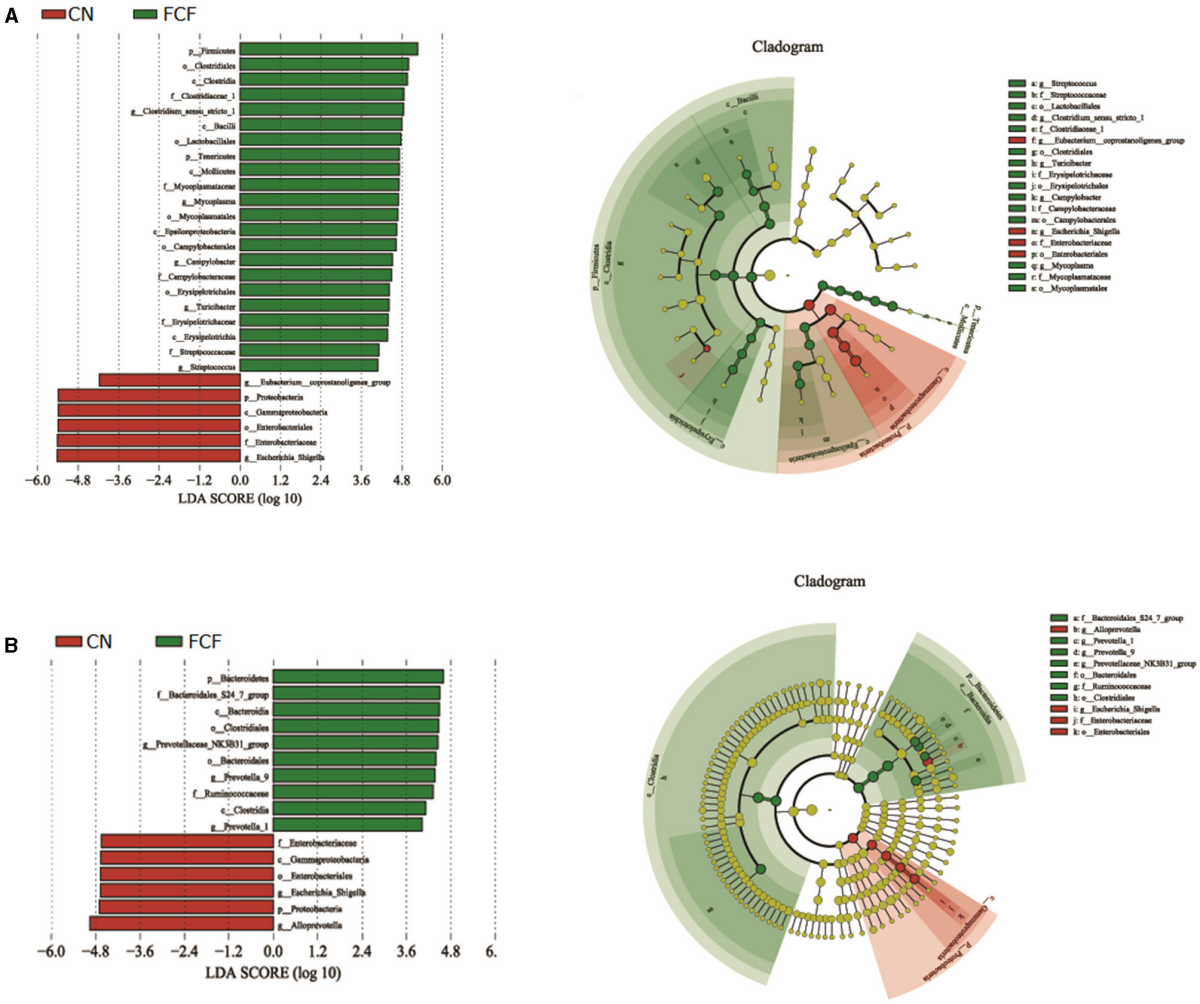
LEfSe analysis filtered out the biomarkers of the microbial community after pigs received fermented complete feed (*n* = 3). **(A)** LEfSe analysis of jejunum content, **(B)** LEfSe analysis of cecum content. CN, control group (pigs received a basal diet); FCF, fermented complete feed group (pigs received fermented complete feed).

The LEfSe was used to identify bacterial biomarkers that were associated with fermented treatment. It showed that in jejunum content, the CN group was associated with the increased relative abundances of *Proteobacteria* at the phylum level, and *Eubacterium_coprostanoligenes_group* and *Escherichia_Shigella* at the genus level, whereas two phyla (*Firmicutes, Tenericutes*) and five bacterial genus (*Clostridium_sensu_stricto_1, Mycoplasma, Campylobacter, Turicibacter*, and *Streptococcus*) were the biomarker in the FCF group (Figure 6A). In the cecum content, the CN group was associated with the increased relative abundances of *Proteobacteria* at the phylum level, *Escherichia_Shigella* and *Alloprevotella* at the genus level, the FCF group was associated with the increased relative abundances of *Bacteroidetes* at phylum level, and *Prevotellaceae_NK3B31_group, Prevotella_9*, and *Prevotella_1* at the genus level (Figure 6B).

## Discussion

MFF has been widely investigated to reduce the use of antibiotic growth promoters in pig production (25, 26). Previous studies had confirmed the growth-promoting effects of fermented liquid feed in pig production (27, 28). However, literature on solid-state FCF is lacking. In the present study, a mixed culture containing *Lactobacillus, C. utilis, B. subtilis*, and *A. niger* was used for producing FCF. Previous studies have demonstrated that nutritional components were changed before and after fermentation (29, 30). In the present study, the nutritive values were also changed in a certain extent, for example, the CP content increased to 16.92% (basal feed is 15.89%), and crude fiber decreased to 3.27% (basal feed is 3.45%). The results from the present study showed that the ADG and ADFI were significantly increased after pigs received FCF, and the F/G ratio was significantly decreased compared with pigs fed with basal diet, which consist of, in previous studies, pigs that received fermented liquid feed (27, 28). It indicated that solid-state FCF has potential to be efficient in promoting animal growth without antibiotic use. The reasons of FCF to promote growth performance of pigs in this study may be due to the following characteristics gained from solid-state fermentation. First, *A. niger* has strong enzyme (cellulase, protease, and amylase)–secreting ability during fermentation (13), after fermentation, the macromolecules were decomposed into small molecules, which improves the palatability and nutrient digestibility of feed. Second, FCF contains *Lactobacillus* (1.5 × 10^8^ CFU/g), *C. utilis* (2.1 × 10^6^ CFU/g), and *B. subtilis* (3.0 × 10^8^ CFU/g), which can function as probiotics to promote growth performance (1, 31, 32).

Evidence shows that serum biochemical parameters reflect comprehensive functions of body organs and nutritional metabolism, which could indirectly response the health status of pigs (33, 34). Serum ALP and GLU index can reflect the growth and health of the animals (35–38). In the present study, pigs fed with FCF had higher ALP, GLU, IgG, IgM, SOD, and T-AOC levels compared with pigs that received basal feed, which demonstrated that FCF has benefit for animal growth, nutrition absorption, and animal health. Similar results have been obtained in other studies using fermented feed (39–41). Health benefit effects of FCF may be related to probiotics contained in fermented feed. Because probiotics such as *B. subtilis* has earned many benefit claims, including the immune modulation, promotion of nutrients, and digestibility, along with improvements of intestinal health and growth performance in animals (42, 43). Therefore, FCF may be an important nutritional strategy to maintain the health status of pigs.

Microbial fermentation has been recognized as an effective way to improve nutritional composition and decrease the antinutritional effects of feed ingredients (44–46), which had a benefit to improve carcass traits and meat quality. In the present study, the fermented diet significantly increased carcass length and significantly decreased the average back-fat thickness. Similar results were obtained by Qiu et al. (47), who reported that the fermented diet significantly increased the loin eye area and lean mass percentage of finishing pigs. However, the mechanisms underlying this observation remain unknown. The possible reasons of FCF to promote carcass traits may be related to increased AA profile of the feed after fermentation (47), which may play an important role in muscle accretion through increased protein synthesis signaling and suppressed degradation signaling in LDM in finishing pigs (47, 48). The pH value, drip loss, CP content, and fat content are commonly used indices for evaluating meat quality of pork (34, 49, 50). pH is an important indicator of meat quality as it is related to the shelf life, color, and water-holding capacity of the meat (51). In the present study, we found that FCF has no effects on pH value, which indicated that FCF would not affect the shelf life, color, and water-holding capacity of meat. Drip loss reduces meat nutritional value by carrying away some nutrients in exudates and leading to drier and tougher meat and with a worse flavor (49). The results of this study showed that FCF would not cause muscle drip loss, which means that pigs fed with FCF would not affect meat nutritional value. Meat is the main protein source for human beings with high economic value. In the present study, the CP content was increased significantly after the pigs received FCF, which indicated that FCF had a beneficial effect on meat quality. The intramuscular fat content is also an important index for evaluating meat quality and is important for tenderness, succulence, and flavor (34, 50). In the present study, there was no difference of fat content between the CN and FCF groups, which meant that there was no adverse effect on fat content in pork when pigs were fed with FCF. These results likely were because *Lactobacillus, C. utilis*, and *B. subtilis* had prebiotic effects on improving pork quality such as pH value, drip loss, CP content and fat content.

The AA profile in pork is important to evaluate meat quality. The type, content, and proportion of AAs in pork are related to meat quality and meat flavor. For example, essential AA (EAA) determines the quality of muscle protein quality, and the delicious AA, such as Ala, Gly, Glu, Asp, and Ser, may affect the delicate flavor of pork (34, 51). In the present study, the higher Lys and Glu content in pork from pigs fed with FCF indicated that FCF could improve meat quality as well as enhance meat flavor. Because Lys is not only an EAA in the human body, but also an important substance to improve the texture and color and enhance the product yield of meat products (52), and Glu is one of the most important AAs, which is the primary flavor molecule and functions in meat freshness and buffering salty and sour tastes (50).

Fatty acids, including monounsaturated fatty acids (MUFAs), polyunsaturated fatty acids (PUFA), and SFA, are directly related to the nutrient value as well as the basis that constitutes the characteristic flavor of meat (53). MUFA comprised the largest proportion of UFA, with C18:1n9c being the most abundant, and this is consistent with the previous studies in pork (54) and cattle (55). C18:1n9 is suggested to be positively associated with the softness of fat, which could improve the sensory quality of meat (55, 56). PUFA plays a regulatory role on human hormone metabolism and the activity of many enzymes and plays a wide range of roles in regulating lipid metabolism, preventing cardiovascular and cerebrovascular diseases, and delaying the decline of immune function (53). C18:2n6c and C20:4n6 are two important PUFA considered good for human health (57). In the present study, the MUFA (C18:1n9c), PUFA (C18:2n6c, C20:4n6), and total UFA in LDM were enhanced by FCF, which indicated that fermented feed can improve the pork quality by increasing the content of UFA. Excessive intake of SFA would increase the risk of cardiovascular diseases and easily cause insulin resistance (58, 59). Thus, SFA can be considered as an important index of pork quality. The present results showed that pigs that received FCF significantly decreased the C18:0 and the total SFA content. These data suggested that FCF could improve the composition of muscle fatty acids by increasing the content of UFA and decreasing the content of SFA, thus improving pork quality. The composition of the fatty acid profile significantly affects the fatty acid composition of the meat (60). Thus, the mechanisms of FCF, which improved fatty acid composition of pork, are likely because the composition of fatty acids in feed was improved after fermentation.

Previous studies have reported the beneficial effects of fermented feed on the swine gut microbiota during different growth states (61–63). Alpha diversity has been considered to be a comprehensive indicator of species richness and evenness (64). In the current study, the ACE index, Chao1 index, Shannon index, and Simpson index were calculated to assess the diversity, richness, and phylogenetic diversity of the bacterial community of jejunum and cecum content. The results showed that FCF significantly increased the Shannon index and Simpson index in jejunum and significantly increased the ACE index, Chao1 index, Shannon index, and Simpson index in cecum. Bata diversity analysis showed that FCF had a scattered distribution structure in both jejunum content and cecum content. It indicated that FCF could increase the diversity of gut microbes. It not only influenced the diversity of gut microbes, but also FCF affected the gut microbiome composition. Generally, *Firmicutes, Bacteroides, Proteobacteria*, and *Fusobacteria* are the dominant phyla of mammalian gut (65, 66). In the present study, *Proteobacteria, Firmicutes, Tenericutes*, and *Bacteroidetes* were the dominant phyla in jejunum and cecum contents. Pigs fed with FCF, the *Proteobacteria* phylum was significantly decreased in jejunum and cecum, *Firmicutes* and *Tenericutes* were significantly increased in jejunum, and *Bacteroidetes* and *Tenericutes* were significantly increased in cecum. Similarly, Xie et al. (67) showed that weaned pigs fed with fermented soybean meal significantly increased the proportion of *Firmicutes* but decreased the proportion of *Proteobacteria*. *Proteobacteria* mainly includes gram-negative bacteria and composed of many pathogenic microorganisms, such as *Escherichia*, which is associated with intestinal inflammation (67, 68). *Firmicutes* mainly includes gram-positive bacteria, such as *Lactobacillus* and *Clostridium*, which is benefit for animal health (69, 70). Accompanying the higher proportion of *Firmicutes* and lower proportion of *Proteobacteria*, the pigs fed FCF showed a better growth performance, indicating a possible relationship between the composition of bacteria and BW gain. At the genus level, pigs fed with FCF significantly increased *Clostridium* genus abundance and significantly decreased *Escherichia–Shigella* genus abundance. Many bacteria in *Clostridium* genus are thought to be probiotics, such as *Clostridium butyricum*, which had been indicated to improve growth and maintain intestinal barrier function of pigs (71, 72). Some bacteria in *Escherichia–Shigella* are known to cause intestinal pathology in pigs; the increased *Escherichia–Shigella* abundances may cause diarrhea of pigs (70, 73), which may cause adverse effects on animal health. These results are likely because *Lactobacillus, C. utilis*, and *B. subtilis* had prebiotic effects on improving intestinal microbiome composition.

The current study showed that FCF could improve the pig health status by modulating gut microbiota, whereas the growth of pigs and pork quality are closely related to pig health. Gut microbiota plays a vital role in host health, which is thought to tightly associate with the intestinal barrier function including physical barrier, chemical barrier, immune barrier, and microbial barrier (74). Thus, we can speculate that FCF promotes the growth performance, carcass performance, and meat quality of finishing pigs, maybe through modulating gut microbiome composition. Of course, this hypothesis needs further confirmation.

## Conclusions

**Figure 7:**
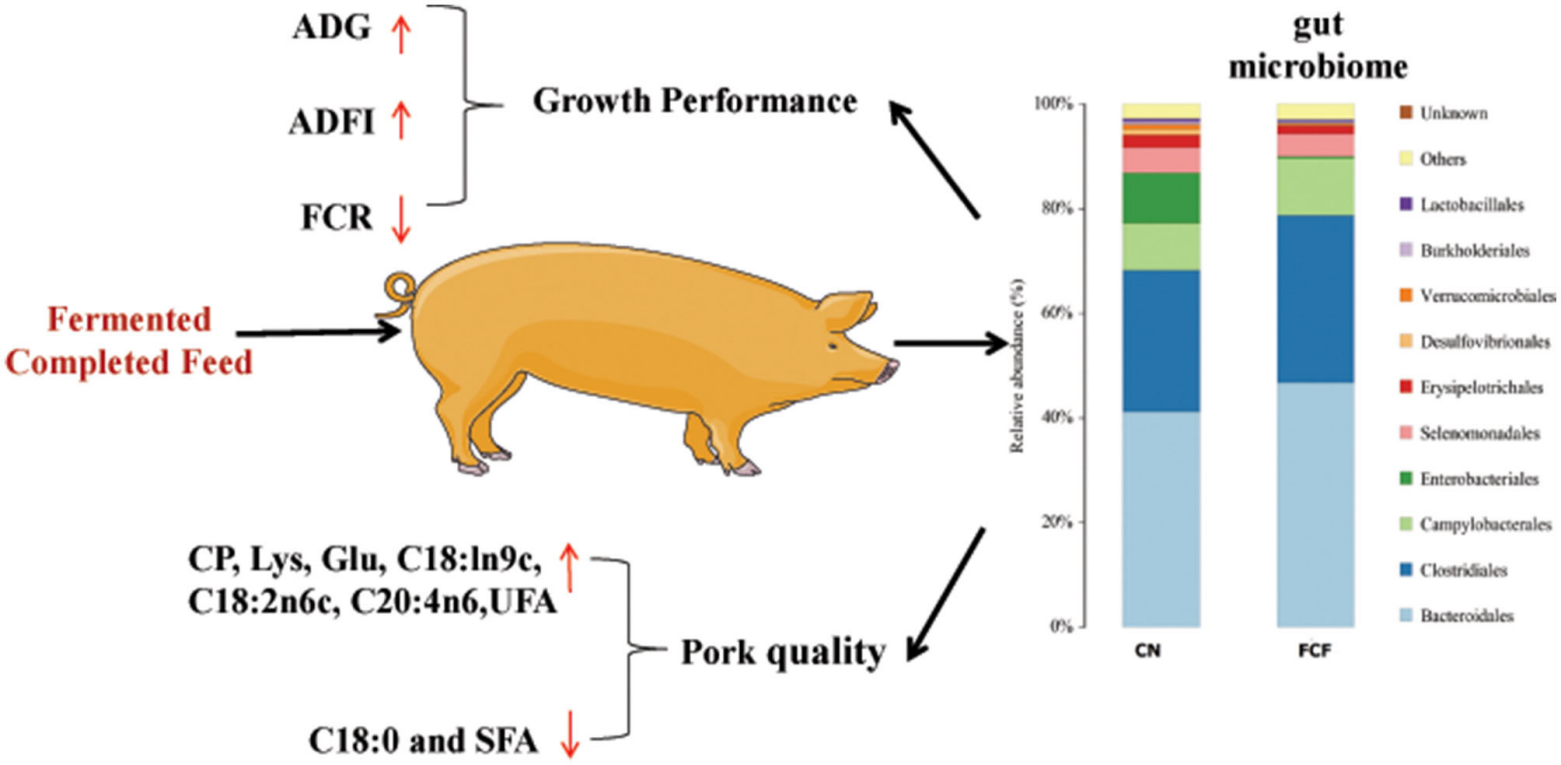
Fermented complete feed promotes the growth performance and pork quality may be through modulating gut microbiome composition. CN, control group (pigs received a basal diet); FCF, fermented complete feed group (pigs received fermented complete feed); ADG, average daily gain; ADFI, average daily feed intake; FCR, feed conversion ratio; CP, crude protein; Glu, glutamine; C18:0, stearic acid; C18:1n9c, oleic acid; C18:2n6c, linoleic acid; C20:4n6, arachidonic acid; UFA, unsaturated fatty acids; SFA, saturated fatty acids.

The current study showed that the ADG and ADFI of pigs significantly increased and the F/G ratio significantly decreased after the pigs received FCF; the ALP, GLU, IgG, IgM, SOD, and T-AOC content significantly increased after the pigs received FCF; pigs that received FCF had a higher carcass length, crude protein content, lysine content, Glu content, C18:ln9c, C18:2n6c, C20:4n6, and unsaturated fatty acids and had a lower average back-fat thickness, C18:0, and saturated fatty acids; FCF significantly reduced the abundances of pathogenic bacteria *Proteobacteria* phylum and *Escherichia–Shigella* genus and enhanced the abundances of beneficial bacteria *Firmicutes* phylum and *Clostridium* genus (summarized in Figure 7). In summary, FCF had a certain effect on the improvement of growth performance serum biochemical profile, carcass traits, meat proximate composition, AA and fatty acid composition, and gut microbiome composition of finishing pigs. The results of the present study thereby can provide some references for the application of FCF in animal production.

## Data Availability Statement

The datasets presented in this study can be found in online repositories. The names of the repository/repositories and accession number(s) can be found below: https://www.ncbi.nlm.nih.gov/, PRJNA725094.

## Ethics Statement

The animal study was reviewed and approved by the Animal Welfare Committee of the Shanxi Agricultural University (Taigu, China). The ethic approval number is SXAU-2018-0093.

## Author Contributions

XT and KZ performed the experiments, contributed to experimental concepts and design, provided scientific direction, and finalized the manuscript. XT, XL, and KZ performed the statistical analyses and wrote the manuscript. All authors read and approved the final manuscript.

## Funding

This research was funded by grants from the World Top Discipline Program of Guizhou Province (No. 125 2019 Qianjiao Keyan Fa); the Key Science and Technology Program of Guizhou Provence (No. 5411 2017 QianKehe Pingtai Rencai); China Overseas Expertise Introduction Program for Discipline Innovation (D17016); the Key Research and Development Program of Shanxi Province (201603D221026-4); the Central Guide Local Science and Technology Development Special Fund (2017GA630002); the Shanxi Academy of Agricultural Sciences Director and Youth Guidance Special Item (yydzx14); the Natural Science Research Project of Education Department of Guizhou Province (Qianjiaohe KY Zi [2021] 294); and the Doctoral Launched Scientific Research Program of Guizhou Normal University (GZNUD [2018]26).

## Conflict of Interest

The authors declare that the research was conducted in the absence of any commercial or financial relationships that could be construed as a potential conflict of interest.

## Publisher's Note

All claims expressed in this article are solely those of the authors and do not necessarily represent those of their affiliated organizations, or those of the publisher, the editors and the reviewers. Any product that may be evaluated in this article, or claim that may be made by its manufacturer, is not guaranteed or endorsed by the publisher.
